# Intraoperative Cell Savage, Infection and Organ Failure in Infective Endocarditis Patients—A Retrospective Single Center Evaluation

**DOI:** 10.3390/jcm12010382

**Published:** 2023-01-03

**Authors:** Christoph Sponholz, Oliver Sommerfeld, Caroline Moehl, Thomas Lehmann, Marcus Franz, Michael Bauer, Torsten Doenst, Gloria Faerber, Mahmoud Diab

**Affiliations:** 1 Department of Anesthesiology and Intensive Care Medicine, Jena University Hospital, Friedrich Schiller University Jena, 07743 Jena, Germany; 2 Institute of Medical Statistics, Computer and Data Sciences, Jena University Hospital, Friedrich Schiller University Jena, 07743 Jena, Germany; 3 Department of Internal Medicine I, Jena University Hospital, Friedrich Schiller University Jena, 07743 Jena, Germany; 4 Clinic for Cardiothoracic Surgery, Jena University Hospital, Friedrich Schiller University Jena, 07743 Jena, Germany

**Keywords:** infective endocarditis, organ failure, cell salvage, blood transfusion

## Abstract

Surgery is indicated in about 50% of infective endocarditis patients, and bleeding or the transfusion of blood a common finding. The intraoperative use of cell salvage may reduce the perioperative transfusion requirement, but its use is limited in the underlying disease. In this retrospective study, we therefore evaluated *n* = 335 patients fulfilling the modified Duke criteria for infective endocarditis characterized by the use of intraoperative cell salvage with autologous blood retransfusion. Inflammation markers and organ dysfunction, including catecholamine dependency, were evaluated by using linear regression analysis. Between 2015 and 2020, 335 patients underwent surgery for left-sided heart valve endocarditis. Intraoperative cell salvage was used in 40.3% of the cases, especially in complex scenarios and reoperation. Intraoperative cell salvage significantly altered the white blood cell count after surgery. On average, leucocytes were 3.0 Gpt/L higher in patients with intraoperative cell salvage compared to patients without after adjustment for confounders (95% CI: 0.39–5.54). Although the difference in WBC was statistically significant, i.e., higher in the ICS group compared to the no-ICS group, this difference may be clinically unimportant. Organ dysfunction, including hemodynamic instability and lactate values, were comparable between groups. In conclusion, intraoperative cell salvage enhanced the re-transfusion of autologous blood, with minor effects on the postoperative course of inflammatory markers, but was not associated with increased hemodynamic instability or organ dysfunction in general. The restriction of intraoperative cell salvage in surgery for infective endocarditis should be re-evaluated, and more prospective data in this topic are needed.

## 1. Introduction

The use of intraoperative cell salvage (ICS) was shown to reduce perioperative transfusion requirements in multiple clinical scenarios [1]. Cardiac surgical procedures account for a significant amount of allogeneic blood transfusion, and, accordingly, ICS was shown to reduce the transfusion rates of red blood cells without an adverse impact on clinical outcomes [2,3]. Therefore, ICS is an integral part of the patient blood management concept in cardiac surgery [4,5,6]. However, the use of ICS is not recommended in patients suffering from systemic infection or in circumstances where a wound blood might be contaminated [7].

Infective endocarditis (IE) represents a life threatening disease with in-hospital mortality rates of approximately 40% [8]. Cardiac surgery is necessary in almost half of patients with IE [9] and is frequently more complicated than cardiac surgery for non-endocarditis valve pathologies [10]. For patients undergoing surgery for IE, the most common reasons for the complexity of surgery are previous cardiac surgery, in 48% [11], multiple valve surgery, in 38%, and the presence of a cardiac abscess, in 27%. In addition to endocarditis-related coagulopathy, the complexity of IE surgery may lead to increased perioperative bleeding and consequently higher requirements for transfusion of blood products, especially in patients with prosthetic valve IE [12]. 

It is therefore reasonable to include ICS in the concept of cardiac surgery for IE, especially in the case of reoperation or prosthetic valve surgery. On the other hand, the perioperative course of IE patients is often accompanied by sepsis or septic shock, leading to multiorgan failure with high mortality rates [13]. In these circumstances, the use of ICS is restricted by guidelines for transfusion [14], although the data supporting these findings are limited.

The debate about using or not using ICS in complex IE surgery is a daily praxis in cardiac surgery centers. This study aims to elucidate the current practice of ICS use in our center and to present data on ICS use among IE patients with a focus on inflammatory parameters and organ failure in the early postoperative period.

## 2. Materials and Methods

### 2.1. Patient Recruitment and Study Design

The study was approved by the ethical committee of Friedrich-Schiller University Jena, Germany (registration number: 2021-2502-Daten, Chairperson: Prof. E. Schleussner) on 4 January 2022. Informed consent was waived because of the anonymous and observational character of the study. All charts from patients operated for left-sided infective endocarditis between 2015 and 2020 at our center were reviewed. Patients were divided into two groups according to the intraoperative usage of cell salvage.

Within the study period, *n* = 335 patients underwent cardiac surgery for left-sided endocarditis and were eligible for evaluation.

### 2.2. Inclusion Criteria

Only patients fulfilling the modified Duke criteria for the definition of infective endocarditis were included in our evaluation [15]. Patients with possible IE prior to surgery were not included in the analysis.

### 2.3. Intraoperative Cell Salvage Use

ICS usually consists of three components: (1) the collection of tissue blood into a collection reservoir. In this step, tissue blood is usually mixed with heparinized saline to avoid clotting, and tissue debris is removed through a filter membrane. (2) The separation and washing of eryhtrocytes in a centrifugation process with normal saline solution. In this step, the effluent containing plasma fractions, platelets, WBC, free hemoglobin and anticoagulats (i.e., heparin) is separated and discarded. (3) The collection of the washed erythrocyte solution for re-transfusion. A hematocrite of >50% and protein reduction of >90% represent the quality standard of ICS blood and is recommended. However, washed ICS blood may not be immunologically inert, and contamination with gram-positive bacterial commensals of the skin were described without adverse events in the affected patients. Therefore, recent guidelines do not recommend ICS in the cases of infection or tissue contamination.

**Figure 1:**
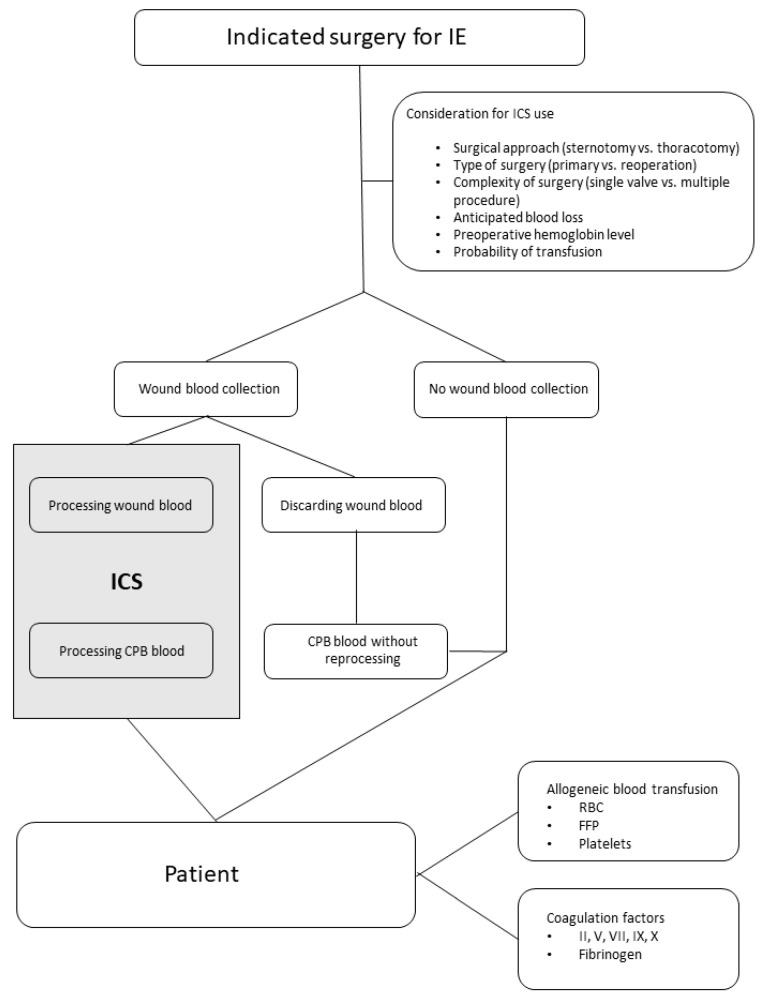
Flowchart highlighting the decision pathway for ICS use and transfusion during IE surgery. IE: infective endocarditis; ICS: intraoperative cell salvage; CPB: cardiopulmonary bypass; RBC: red blood cells; FFP: fresh frozen plasma.

In the current setting, ICS was implemented on the discretion of the cardiac surgeon and the anesthesiologist in charge following the flowchart depicted in Figure 1. ICS was performed with the intraoperative autotransfusion system XTRA (LivaNova GmbH, Munich, Germany). The system was built up and used according to the manufacturer’s instructions. To avoid clotting, the collection system was primed with normal saline solution and an additional supplementation of 25,000 IE of heparin per liter NaCl, as recommended.

ICS was begun after sternotomy during the preparation process and the prior implementation of the cardiopulmonary bypass circuit. After CPB initiation, tissue blood was preferentially suctioned into the CPB reservoir. Once the infected tissue areas were prepared, blood and tissue debris were discarded via an external suction unit to avoid the re-transfusion of potentially affected and contaminated material. Blood collection via ICS was restarted after CPB separation to minimize blood loss during the restoration of coagulation and chest closure. After weaning from the CPB, residual circuit blood was preferentially processed within the ICS procedure to remove residual heparin and to concentrate hemoglobin content of the machine blood. The ICS-processed blood was administered before transferring the patient to the ICU. Additional blood products or coagulation factors were transfused as needed. The ICS procedure is also depicted in the flowchart of Figure 1.

### 2.4. Biochemical and Laboratory Markers

Data were obtained from electronic patient charts (COPRA, version 6.78.2.0 and 5.24.974; COPRA System GmbH, Sasbachwalden, Germany) and the clinical database (SAP, version 7300.1.3.1079, Walldorf, Germany). Biochemical values were taken prior to surgery, immediately after surgery and on the first three postoperative days. To describe the use of vasoactive and inotropic agents, the vasoactive-inotropy score (VIS) [16] was calculated as recommended. Organ dysfunction was defined by calculating the Sequential Organ Failure Assessment [17].

### 2.5. Statistical Analysis

Whereas continuous data are presented in median [25th–75th percentile] values, categorical data are displayed as number and percentage. Continuous patient characteristics were compared by two-sided non-parametric Mann–Whitney U test, and categorical data were compared by two-sided chi-square test. Independent risk factors associated with the referring laboratory and clinical markers were evaluated by applying binary logistic regression analysis. Regression coefficients with 95% confidence intervals as well as partial eta squared were reported to assess the effect of the risk factors. *p* values < 0.05 were considered to be statistically significant, all analyses were exploratory and no correction for multiple testing was performed. Statistical analyses were performed using IBM SPSS Statistics, Version 26.0 (IBM Corporation, Armonk, NY, USA), and the graphics were designed with SigmaPlot, Version 14.5 (Systat software, Erkrath, Germany).

## 3. Results

### 3.1. Patient Characteristics

In the period between January 2015 and December 2020, we identified *n* = 335 patients who underwent surgery for left-sided IE at our institution. Patients had a median age of 67 [58.0–75.0] years and the majority were male (76%). The median EuroSCORE II prior to surgery was 6.3 [3.45–10.63]. The median duration of surgery was 200 [154.0–269.0] min, with an intraoperative time on cardiopulmonary bypass of 113 [83.0–156.0] min and a cross clamping time of 70 [59.0–98.0] min. The most common surgical procedures were complex procedures (including multiple valve procedures with or without the resection of an abscess or aortic root) (46.9% of cases), followed by single aortic valve replacement via sternotomy (30.4%), single mitral valve replacement/reconstruction (13.7%) or minimal invasive mitral valve surgery (9%). The rate of reoperation for prosthetic IE was 29.6%. Intraoperative cell salvage was used in *n* = 135 (40.3%) of the cases. ICS was more common in complex surgical cases as well as reoperations. Duration of surgery, time on CPB as well as cross clamping time were longer in patients with ICS compared to the no-ICS patients.

All patient characteristics are listed in Table 1.

### 3.2. Course of Inflammatory Markers

**Figure 2:**
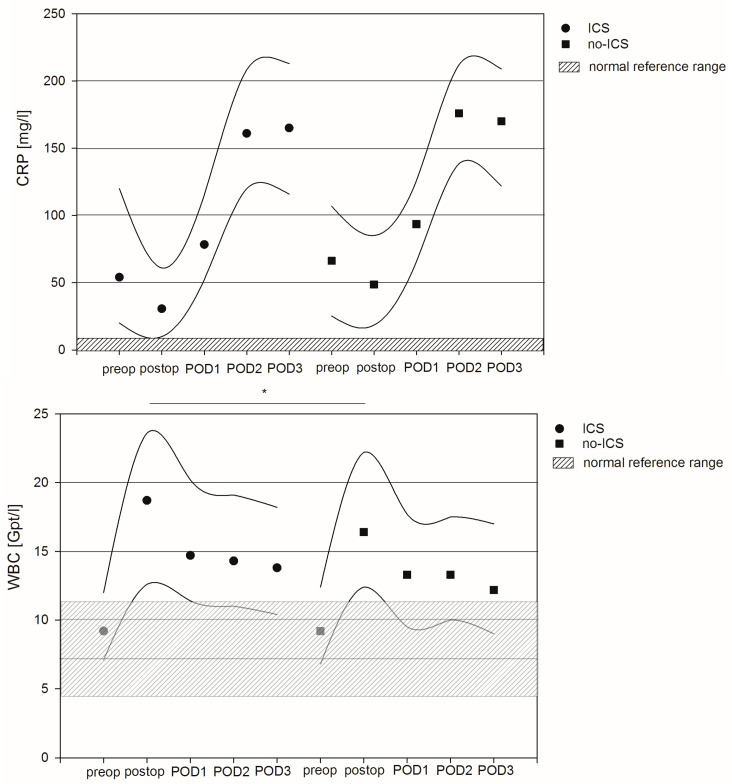
Course of inflammatory markers C-reactive protein and white blood cell count between intraoperative cell salvage (ICS) and no-ICS patients. Circles and squares represent median values, and lines mark 25th and 75th percentiles. * indicates significant difference between the groups resulting from linear regression analysis.

Median values of C-reactive protein (CRP) were preoperatively elevated in both patient groups and further increased in the postoperative phase, with peak values on postoperative day two (POD2). In contrast, white blood cell counts (WBCs) were in the normal range prior to surgery and increased in the postoperative phase. Peak values appeared immediately after surgery and declined within the next three days, with values staying above the normal upper value. Linear regression analysis revealed no significant differences in terms of CRP prior to or after surgery between ICS and no-ICS patients. However, the WBC values were significantly higher immediately after surgery in ICS patients compared to no-ICS patients (*p* = 0.024). On average, the WBC values of ICS patients were 3.0 Gpt/l higher compared to no-ICS patients after adjustment for confounders (95% CI: 0.39–5.54). Furthermore, reoperation, former values of inflammation and lactate values, as well as markers of organ dysfunction, were also independent factors associated with differences in terms of CRP and WBC values in the perioperative phase. For details, see Figure 2 and Tables S1 and S2 of the Supplementary Materials.

### 3.3. Course of Hemodynamic Parameters

**Figure 3:**
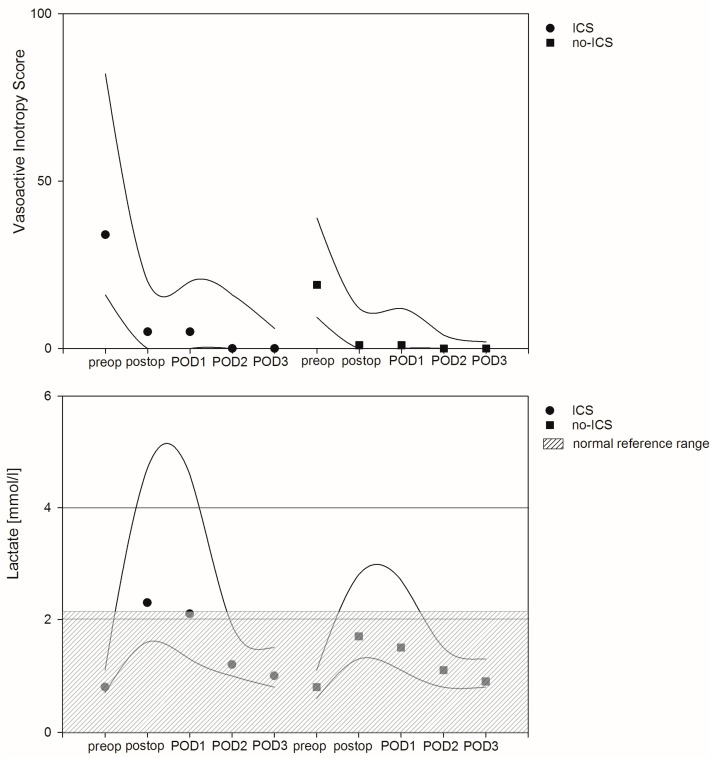
Course of vasoactive inotropy score and lactate levels as surrogate for hemodynamic instability between intraoperative cell salvage (ICS) and no-ICS patients. Circles and squares represent median values, and lines mark 25th and 75th percentiles.

Median values in terms of the vasoactive inotropy score (VIS) peaked intraoperatively and continuously declined within the first three days after surgery. Linear regression analysis found no significant differences between both patient groups. However, the intraoperative lactate level, perioperative renal dysfunction (defined by elevated creatinine) and the grade of inflammation (defined as elevated CRP values) were factors associated with hemodynamic instability. In detail, VIS in patients with elevated preoperative lactate levels were on average 19.7 points higher compared to patients with low preoperative lactate values after adjustment for confounders (95% CI: 8.3–31.2, *p* = 0.001). Intraoperative VIS in patients with preoperative high creatinine levels were on average 0.3 points higher compared to patients without elevated creatinine levels after adjustment for confounders (95% CI: 0.15–0.45, *p* = 0.001). Finally, intraoperative VIS in patients with elevated CRP values were on average 0.3 points higher compared to patients with lower CRP values after adjustment for confounders (95% CI: 0.08–0.44, *p* = 0.005). Median lactate values peaked immediately after surgery and declined over the following postoperative days. Again, ICS was not associated with increased perioperative lactate values, but linear regression analysis revealed the perioperative use of catecholamines, the duration of the CPB and cross clamping time and former lactate values as factors associated with the course of lactate in the referring patient cohort (Figure 3 and Tables S1 and S2 of the Supplement).

### 3.4. Perioperative Organ Dysfunction

**Figure 4:**
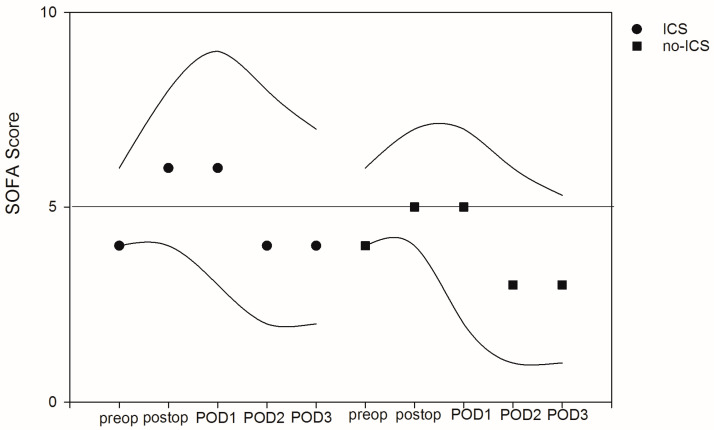
Course of sequential organ failure assessment (SOFA) between intraoperative cell salvage (ICS) and no-ICS patients. Circles and squares represent median values, and lines mark 25th and 75th percentiles.

To describe the course of organ dysfunction, the SOFA score was calculated daily. The median preoperative SOFA was 4 [4.0–6.0], with peak values on the day of surgery. There were no significant differences in terms of the referring SOFA scores. The factors associated with organ dysfunction were the grade of inflammation (postoperative SOFA with elevated WBCs were on average 0.1 point higher (95% CI: 0.02–0.145, *p* = 0.010) after adjustment for confounders), the patient’s age (postoperative SOFA increased on average by 0.05 point with every year of age (95% CI: 0.01–0.08, *p* = 0.013) after adjustment for confounders) and preexisting organ failure (postoperative SOFA increased on average by 1 point with every point increase in preexisting SOFA score (95% CI: 0.05–1.14, *p* = 0.001) after adjustment for confounders). A separate evaluation of the underlying laboratory markers predicting organ function showed values above the upper normal range for creatinine and bilirubin as well as values on the lower limit of normal for platelets on POD1 without any differences between ICS and no-ICS patients (see Figure 4 and Tables S1 and S2 of the Supplement).

### 3.5. Intraoperative Transfusion Requirement

Patients in the ICS group were more likely to receive the transfusion of red cells and coagulation products while undergoing surgery. In detail, patients in the ICS group received more packed red blood cells (3 [2.0–5.0] vs. 2 [0.0–3.0], *p* = 0.001), fresh frozen plasma (0 [0.0–600.0] vs. 0 [0.0–0.0] mL, *p* = 0.001), coagulation factors II, V, VII, and X (2000 [0.0–3000.0] vs. 0 [0.0–2000.0], *p* = 0.001) IU, fibrinogen (2 [2.0–4.0] vs. 0 [0.0–2.0] g, *p* = 0.001) and platelets (1 [0.0–2.0] vs. 0 [0.0–0.0] units, *p* = 0.001) compared to patients in the no-ICS group. Moreover, patients in the ICS group additionally received in median 700 [415.5–1000.0] mL of washed cell salvage blood. Median hemoglobin levels were comparable in the perioperative phase in both patient groups.

### 3.6. Patient Outcome Data

Patients stayed in hospital for a median 14 [8.0–22.0] days, and a median of 6 [3.0–13.9] days on ICU after surgery. The postoperative ventilation time was a median of 14 [6.1–68.4] h after surgery. The days on hemodialysis or circulatory support after surgery were a median of 0 [0.0–2.0] days or 0 [0.0–0.0] days, respectively. The hospital survival rate was 69.6% in the total patient cohort (see Table 2).

## 4. Discussion

The aim of this retrospective study was to evaluate the course of parameters associated with the intraoperative use of cell salvage in patients undergoing surgery for infective endocarditis. The results of the study can be summarized as follows:

1. ICS was frequently used in surgery for IE, especially in complex surgical cases and reoperation.
2. ICS did not increase inflammation, except for WBCs immediately after surgery. However, the difference in terms of WBC was statistically significantly higher in the ICS group compared to the no-ICS group, and this difference may be clinically unimportant.
3. ICS did not alter the course of hemodynamic instability, defined by catecholamine dosage and lactate values.
4. Patients with IE usually present with varying degrees of organ dysfunction in the perioperative period. ICS did not alter SOFA-related organ dysfunction (i.e., renal–creatinine, liver–bilirubin or coagulation–platelets).
5. Surgery for IE was associated with a high probability of a transfusion requirement. Due to more complex cases and the resulting surgical approaches, patients in the ICS group were more likely to be transfused with RBC or coagulation products. The use of ICS led to a significant amount of washed tissue blood for re-transfusion.

ICS was designed to collect tissue blood during surgery associated with moderate to high blood loss. During the washing process, tissue debris and other agents are removed, while patient’s erythrocytes are collected for re-transfusion [18]. Recent meta-analyses describe a reduction in the transfusion probability of 39% by using ICS, especially during orthopedic and cardiac surgery [19,20]. Therefore, ICS is an integral part of the patient blood management concept [4]. Although there is no absolute contraindication, the use of ICS in infected and contaminated fields remain controversial. Therefore, recent guidelines recommend the application of ICS in these circumstances on a case-to-case basis and to consider its use with caution [21]. However, data regarding the benefits or disadvantages are scarce [22] and are to the best of our knowledge non-existent for IE patients.

In theory, ICS may transfer infective agents and toxins from the surgical field into the patient’s circulation with the aggravation of the inflammatory response and sepsis symptoms. In this context, Bland and colleagues determined the bacterial and endotoxin contamination rate of blood collected during elective cardiac surgery. Blood collected in the cell salvage system was culture positive in 96.8% of the samples and 24% had detectable endotoxin levels. Most of the collected blood contained gram-positive bacterial commensals of the skin. However, none of the patients presented with adverse events after surgery [23]. In another study, Luque-Oliveros was able to detect bacterial species in 85% of the red blood cell reinfusion bag of the cell salvage system, with staphylococcus epidermidis (69%) being the most frequent microorganism. Staphylococcus epidermidis was most likely found in patients with a high body mass index and valve surgery [24]. In neither of these studies were adverse events recorded in the patient’s clinical course or outcome. Cardiac surgery with the subsequent use of cardiopulmonary bypass and suction and the retransfusion of cardiotomy suction blood significantly elevates profinflammatory cytokines in the postoperative phase. Avoiding the retransfusion of cardiotomy suction blood reduces the postoperative inflammatory release of TNF-alpha, IL-6 and complement factor 3a [25]. In this respect, ICS was shown to reduce proinflammatory mediators, such as cytokines or complement system components, in comparison to direct cardiotomy suction [3]. Damgaard and colleagues showed reduced plasma IL-6 and IL-8 levels after ICS use in the immediate postoperative phase in elective CABG patients. Moreover, tumor necrosis factor receptor, IL-10 and procalcitonin levels were significantly reduced in ICS blood [26]. Furthermore, even in off-pump coronary artery bypass grafting surgery, proinflammatory cytokines were elevated in the postoperative phase and ICS was able to remove cytokines effectively [27]. Taken together, cardiotomy suction blood and processed ICS blood is frequently contaminated by bacterial commensals. Moreover, the surgical procedure itself and the use of CPB circuits enhances the inflammatory response, leading to elevated levels of cytokines and complement factors in the postoperative phase. ICS is able to reduce the levels of cytokines from cardiotomy suction blood. In a previous study, we demonstrated enhanced proinflammatory markers in IE patients compared to non-IE patients immediately after CPB and within 6h of surgery [28]. Moreover, Träger and colleagues described peak values in terms of IL-6 and IL-8 levels in IE patients during and immediately after surgery [29]. However, data on the inflammatory profile or bacterial contamination of ICS-processed blood in IE patients are missing.

Hemodynamic instability, defined by elevated lactate levels and catecholamine support, is common during the perioperative phase in patients with IE [29,30,31]. Lactate levels and catecholamine support in our patient cohort peaked in the early postoperative phase and continuously declined in the days after surgery. These data are in line with previous reports [29,31]. Belletti and colleagues defined factors associated with high-dose inotropic support using vasoactive-inotropic scores of >10. In that case, the duration of surgery, a male gender, the preoperative impairment of kidney function, the worsening of heart failure and the preoperative platelet count presented factors associated with postoperative high-dose inotropic support. Similar data were found in our patient cohort. Here, former levels of lactate and catecholamine support as well as creatinine, bilirubin and CRP levels and the duration of CPB were associated with the course of lactate and catecholamine support. However, ICS was not an independent factor of hemodynamic instability in our patients. Moreover, data supporting hemodynamic alterations of ICS in IE or general surgical patients are missing.

IE is often accompanied by major perioperative complications. Recent data suggest the rate of major complications in IE patients is 38%, with cardiovascular and neurological events as well as renal dysfunction being the most prominent affected organ systems [32]. Moreover, liver dysfunction may play a pivotal role in patients with IE and worsen outcomes [13]. In intensive care patients, the SOFA score was established to describe the degree of organ dysfunction [17]. This score is also frequently used in IE patients and was shown to predict mortality in these patients [33,34]. SOFA scores in our patient cohort peaked on POD1 and declined thereafter. The factors associated with higher SOFA scores were age, the type of surgery, the grade of inflammation (defined by CRP and WBC values), hemodynamic instability (defined by the course of lactate and inotropic support) as well as former course of SOFA values. These data are in line with previous reports that addressed age, surgery, CRP levels and diabetes mellitus as independent factors associated with SOFA scores and mortality in IE patients [33,34,35].

A considerable number of patients undergoing surgery for IE require the transfusion of red packed blood, coagulation products or platelets during the perioperative phase. The probability as well as the amount of the given products increases with the type of surgery and especially in cases of prosthetic valve endocarditis [12]. Patients in the current cohort also received more allogeneic blood transfusions and coagulation products in the context of reoperation. Moreover, ICS was more common in reoperations and complex cases, rather than single valve replacement. With a focus on coagulation, ICS was shown to significantly reduce coagulation factors [36]. Moreover, thrombelastometry fibrinogen levels, measured in FIBTEM-MCF, significantly decreased in the salvaged blood. The authors concluded that amounts of >18.5% of salvaged blood may impair the coagulation function, especially in patients with lower FIBTEM-MCF before and after cardiac surgery [37]. However, the CPB itself alters the coagulation function and hence reduces certain coagulation factors [38]. In this respect, ICS of the residual CPB blood may reduce the incidence of postoperative blood loss and subsequently the application of coagulation products (i.e., fresh frozen plasma) [39]. Furthermore, a considerable number of patients require emergency surgery while being on anticoagulants prior to surgery. Coagulation disturbances in these circumstances are common and need to be managed intraoperatively [40]. On the other hand, IE itself deeply interacts with the coagulation system by increasing systemic coagulation activation, enhancing platelet activity and impairing fibrinolysis [41]. The role of ICS in these circumstances is therefore complex and needs to be characterized in further studies.

To recap, the use of ICS during IE surgery may have advantages as well as disadvantages. Crucial aspects that might be considered prior to ICS use during IE surgery are therefore listed in Table 3.

The current study has several limitations. First, we present a monocentric retrospective evaluation with all its benefits and limitations. Second, ICS was more common in sicker patients with a higher EUROSCORE II prior to surgery, with prosthetic valve IE and reoperation. As these patients usually present with a higher probability of transfusion and coagulation disturbances, and therefore our results may be biased. However, as ICS was more common in these patients, a bias towards the positive effects of ICS is unlikely. Third, due to the retrospective design, we were not able to present data for cytokines or other inflammatory markers related to ICS usage in our patients. Moreover, data on the course of coagulation factors are missing. Therefore, our results should be interpreted with caution and in terms of considerations. However, as we present a large cohort of IE patients with and without ICS use, our data hint towards reliable results with respect to our hypothesis.

## 5. Conclusions

Our results showed that the transfusion of RBC and coagulation factors after surgery for IE was common. The use of ICS enhanced the retransfusion of autologous blood with minor effects on the postoperative course of inflammatory markers. The use of ICS was not associated with increased hemodynamic instability or a worsening of the degree of organ dysfunction, as measured by a SOFA score. The restriction of ICS in IE surgery should be re-evaluated, and more prospective data in this topic are needed.

## Figures and Tables

**Table 1:** Patient characteristics of the total cohort as well as separate values for intraoperative cell salvage (ICS) and no intraoperative cell salvage (no-ICS) use.

	Total	ICS	No-ICS	*p*-Value
	*n* = 335	*n* = 135	*n* = 200	
Age [years]	67 [58.0–75.0]	67 [58.0–75.0]	66.5 [58.0–75.0]	0.741
Male gender, *n* (%)	254 (75.8)	105 (77.8)	149 (74.5)	0.492
EUROSCORE II (%)	6.3 [3.45–10.63]	7.7 [5.34–10.99]	4.7 [2.80–9.04]	0.001
Intraoperative data				
Duration of surgery (min)	200 [154.0–269.0]	255 [211.0–341.0]	172 [138.0.–213.0]	0.001
Time on CPB (min)	113 [83.0–156.0]	141 [108.0–189.0]	96.5 [74.0–131.0]	0.001
Cross clamping time (min)	70 [50.0.–98.0]	88 [62.0–126.0]	61 [47.3–82.8]	0.01
Surgical procedure, *n* (%)				
Complex procedures	157 (46.9)	86 (63.7)	71 (35.5)	0.001
Single aortic, sternotomy	102 (30.4)	35 (25.9)	67 (33.5)	
Single mitral, sternotomy	46 (13.7)	10 (7.4)	36 (18.0)	
Mitral valve, minimal invasive	30 (9.0)	4 (3.0)	30 (9.0)	
Reoperation, *n* (%)				
yes	99 (29.6)	60 (44.4)	39 (19.5)	0.001
no	236 (70.4)	75 (55.6)	161 (80.5)	

**Table 2:** Patient outcome data of the total cohort as well as separate values for intraoperative cell salvage (ICS) and no intraoperative cell savage (no-ICS). ICU: intensive care unit.

Postoperative Outcomes	Total	ICS	No-ICS	*p*-Value
Days in hospital	14 [8.0–22.0]	15 [7.0–23.3]	13 [8.0–21.5]	0.712
Days on ICU	6 [3.0–13.0]	7 [3.0 -14.0]	6 [3.0–13.0]	0.350
Duration of ventilation (h)	14 [6.1–68.4]	20 [7.6–109.6]	12 [5.4–54.7]	0.009
Days on hemodialysis	0 [0.0–2.0]	0 [0.0–3.0]	0 [0.0–2.0]	0.08
Days on mechanical circulatory support	0 [0.0–0.0]	0 [0.0–0.0]	0 [0.0–0.0]	0.162
Hospital survival, *n* (%)	233 (69.6)	86 (63.7)	147 (73.5)	0.177

**Table 3:** Probable advantages or disadvantages of intraoperative cell salvage (ICS) during infective endocarditis (IE) surgery. RBC: red blood cells; CPB: cardiopulmonary bypass.

Advantages of ICS in IE Surgery	Disadvantages of ICS in IE Surgery
Reduction in allogeneic RBC transfusion	Enhanced inflammatory reaction by retransfusion of cytokines
Reduction in cristalloid or colloidal fluid administration	Possible retransfusion of bacteria or bacterial commensals from infected tissue
Reduction in postoperative bleeding tendency by eliminating heparin from CPB blood	Reduction in blood coagulation factors during washing process
Reduction in inflammatory reaction by reducing cytokines from ICS blood	
Reduction in immunologic response against allogeneic blood transfusion	

## Data Availability

Research data supporting this publication are available from the corresponding author.

## Supplementary Materials

The following supporting information can be downloaded at: https://www.mdpi.com/article/10.3390/jcm12010382/s1, Table S1: Course of laboratory and clinical parameters between intraoperative cell saver usage (ICS) and no-ICS patients. Bold mark significant difference, between the groups resulting from linear regression analysis; Table S2: Dependent variables.
