# Supplementary material for: Intraoperative Cell Savage, Infection and Organ Failure in Infective Endocarditis Patients—A Retrospective Single Center Evaluation
Source: J Clin Med. 2023 Jan 3;12(1):382. doi: 10.3390/jcm12010382 (PMC9821775; doi:10.3390/jcm12010382)
Supplement: Supplementary file 1 [file jcm-12-00382-s001.zip › jcm-2099804-supplementary.pdf]

Effect of intraoperative use of cell savage on postoperative infection parameters and organ failure in patients with infective endocarditis – a retrospective single centre evaluation

- Supplemental data -

Christoph Sponholz[1]; Oliver Sommerfeld[1]; Caroline Moehl[1]; Thomas Lehmann[2]; Marcus Franz[3]; Michael Bauer[1]; Torsten Doenst[4]; Gloria Faerber[4]; Mahmoud Diab[4]

1 Department of Anesthesiology and Intensive Care Medicine, Jena University Hospital, Friedrich Schiller University Jena, 07743 Jena, Germany
2 Institute of Medical Statistics, Computer and Data Sciences, Jena University Hospital, Friedrich Schiller University Jena, 07743 Jena, Germany
3 Department of Internal Medicine I, Jena University Hospital, Friedrich Schiller University Jena, 07743 Jena, Germany
4 Clinic for Cardiothoracic Surgery, Jena University Hospital, Friedrich Schiller University Jena, 07743 Jena, Germany

Corresponding author:

Christoph Sponholz

Jena University Hospital,

Department of Anesthesiology and Intensive Care Medicine,
Friedrich Schiller University

Am Klinikum 1

07747 Jena

Germany

Phone: +4936419322225

Fax: +4936419323102

Table S1. Course of laboratory and clinical parameters between intraoperative cell saver usage (ICS) and no-ICS patients. Bold mark significant difference between the groups resulting from linear regression analysis

Parameter	Group	preoperative	postoperative day (values after surgery)	POD1	POD2	POD3
CRP [mg/l]	ICS	54.0 [20.0 - 120.0]	30.6 [9.95 - 60.9]	78.3 [52.2 - 115.3]	161.0 [120.0 - 208.3]	165.0 [116.0 - 213.0]
	no-ICS	66.2 [25.2 - 106.8]	48.6 [18.5 - 85.1]	93.5 [65.7 - 125.9]	176.0 [138.5 - 212.0]	170.0 [122.0 - 209.0]
WBC [Gpt/l]	ICS	9.2 [7.1 - 12.0]	**18.7 [12.6 - 23.6]**	14.7 [11.4 - 20.2]	14.3 [11.0 - 19.1]	13.8 [10.4 - 18.2]
	no-ICS	9.2 [6.8 - 12.4]	**16.4 [12.4 - 22.2]**	13.3 [9.5 - 17.7]	13.3 [10.0 - 17.5]	12.2 [9.0 - 17.0]
VI-Score	ICS	34 [16.0 - 82.0]	5 [0.0 - 20.0]	5 [0.0 - 20.0]	0 [0.0 - 16.0]	0 [0.0 - 6.0]
	no-ICS	19 [9.3 - 39.0]	1 [0.0 - 12.0]	1 [0.0 - 12.0]	0 [0.0 - 4.0]	0 [0.0 - 2.0]
Lactate [mmol/l]	ICS	0.8 [0.7 - 1.1]	2.3 [1.6 - 4.7]	2.1 [1.3 - 4.6]	1.2 [1.0 - 1.9]	1.0 [0.8 - 1.5]
	no-ICS	0.8 [0.6 - 1.1]	1.7 [1.3 - 2.8]	1.5 [1.1 - 2.7]	1.1 [0.8 - 1.5]	0.9 [0.8 - 1.3]
Creatinine [µmol/l]	ICS	94 [70.0 - 132.2]	85 [70.0 - 115.8]	109 [81.0 - 162.5]	114 [73.8 - 179.8]	112 [68.0 - 176.5]
	no-ICS	88 [70.0 - 130.0]	85 [69.0 - 125.5]	105 [73.0 - 154.3]	95 [69.5 - 173.0]	94 [68.0 - 151.0]

Bilirubin [µmol/l]	ICS	11 [7.0 - 18.0]	28 [16.0 - 39.0]	33 [14.0 - 58.0]	19 [9.3 - 46.8]	15 [8.0 - 37.0]
	no-ICS	10 [6.0 - 15.3]	19 [13.0 - 30.5]	17 [10.0 - 40.0]	13 [7.0 - 21.0]	11 [7.0 - 17.0]
Platelets [Gpt/l]	ICS	209 [154.0 - 299.0]	149 [111.0 - 194.5]	143 [100.8 - 202.0]	127 [85.0 - 188.0]	128 [88.0 - 191.0]
	no-ICS	225 [169.0 - 291.0]	166 [127.0 - 229.5]	163 [123.0 - 226.0]	155 [104.5 - 230.0]	145 [106.3 - 225.0]
SOFA	ICS	4 [4.0 - 6.0]	6 [4.0 - 8.0]	6 [3.0 - 9.0]	4 [2.0 - 8.0]	4 [2.0 - 7.0]
	no-ICS	4 [4.0 - 6.0]	5 [4.0 - 7.0]	5 [2.0 - 7.0]	3 [1.0 - 6.0]	3 [1.0 - 5.3]

Table S2. Dependent variables **CRP after surgery**

Parameter	Regressioncoefficient B	Standarderror	T	Sig.	Confidenceintervall		partial Eta-Quadrat
					lower limit	upper limit	
Constant Term	10,719	23,255	0,461	0,646	-35,245	56,682	0,001
ICS - yes	-6,165	5,471	-1,127	0,262	-16,978	4,648	0,009
male gender	7,116	5,980	1,190	0,236	-4,704	18,935	0,010
Reoperation - no	7,602	6,876	1,105	0,271	-5,989	21,193	0,008
Type of surgery - single AV sternotomy	5,262	9,116	0,577	0,565	-12,756	23,280	0,002
Type of surgery - single MV sternotomy	8,095	10,096	0,802	0,424	-11,859	28,048	0,004
Type of surgery - complex case	-5,914	8,842	-0,669	0,505	-23,391	11,562	0,003
Euroscore on admission	-0,142	0,669	-0,212	0,832	-1,465	1,181	0,000
Age at day of surgery	-0,058	0,229	-0,254	0,800	-0,511	0,395	0,000
Hemoglobin [mmol/l] prior to surgery	-2,538	2,596	-0,978	0,330	-7,668	2,593	0,007
Lactate level [mmol/l] prior to surgery	3,648	2,455	1,486	0,140	-1,205	8,501	0,015
procalcotinin level [ng/ml] prior to surgery	0,413	0,630	0,656	0,513	-0,832	1,657	0,003
creatinine level [µmol/l] prior to surgery	0,022	0,031	0,713	0,477	-0,039	0,083	0,003
bilirubin level [µmol/l] prior to surgery	0,423	0,212	1,990	**0,048**	0,003	0,843	0,027
WBC [Gpt/l] prior to surgery	-0,895	0,624	-1,435	0,153	-2,127	0,338	0,014
platelet count [Gpt/l] prior to surgery	0,016	0,025	0,636	0,526	-0,033	0,065	0,003
CRP level [mg/l] prior to surgery	0,638	0,037	17,032	**0,000**	0,564	0,712	0,667

Dependent variable: **CRP POD1**

Parameter	Regressioncoefficient B	Standarderror	T	Sig.	Confidenceintervall		partial Eta-Quadrat
					lower limit	upper limit	
Constant Term	38,259	39,994	0,957	0,341	-40,852	117,370	0,007
ICS - yes	4,709	9,688	0,486	0,628	-14,455	23,873	0,002
male gender	-7,971	10,456	-0,762	0,447	-28,654	12,713	0,004
Reoperation - no	1,194	11,795	0,101	0,920	-22,138	24,526	0,000
Type of surgery - single AV sternotomy	-4,517	15,730	-0,287	0,774	-35,631	26,598	0,001
Type of surgery - single MV sternotomy	1,560	17,175	0,091	0,928	-32,414	35,535	0,000
Type of surgery - complex case	1,514	15,176	0,100	0,921	-28,505	31,533	0,000
Euroscore on admission	-0,760	1,166	-0,652	0,516	-3,066	1,546	0,003
Age at day of surgery	0,047	0,391	0,121	0,904	-0,726	0,821	0,000
Hemoglobin [mmol/l] prior to surgery	1,721	4,560	0,377	0,706	-7,299	10,740	0,001
Lactate level [mmol/l] prior to surgery	-1,741	6,843	-0,254	0,800	-15,277	11,794	0,000
procalcotinin level [ng/ml] prior to surgery	1,206	1,338	0,901	0,369	-1,441	3,853	0,006
creatinine level [µmol/l] prior to surgery	0,156	0,054	2,870	**0,005**	0,049	0,264	0,059
bilirubin level [µmol/l] prior to surgery	-0,294	0,365	-0,805	0,422	-1,017	0,428	0,005
WBC [Gpt/l] prior to surgery	-1,759	1,075	-1,636	0,104	-3,886	0,368	0,02
platelet count [Gpt/l] prior to surgery	0,073	0,044	1,661	0,099	-0,014	0,16	0,02
CRP level [mg/l] prior to surgery	-0,096	0,122	-0,793	0,429	-0,337	0,144	0,005
CRP level [mg/l] after surgery	0,928	0,149	6,233	**0,000**	0,633	1,222	0,227

Dependent variable: **CRP POD2**

Parameter	Regressioncoefficient B	Standarderror	T	Sig.	Confidenceintervall		partial Eta-Quadrat
					lower limit	upper limit	
Constant Term	60,534	47,615	1,271	0,206	-33,756	154,824	0,014
ICS - yes	-17,485	11,615	-1,505	0,135	-40,485	5,515	0,019
male gender	-16,832	12,637	-1,332	0,185	-41,856	8,192	0,015
Reoperation - no	-17,747	13,773	-1,289	0,200	-45,021	9,527	0,014
Type of surgery - single AV sternotomy	36,723	18,916	1,941	0,055	-0,736	74,181	0,031
Type of surgery - single MV sternotomy	31,297	21,108	1,483	0,141	-10,503	73,097	0,018
Type of surgery - complex case	26,471	18,492	1,431	0,155	-10,148	63,090	0,017
Euroscore on admission	0,883	1,420	0,622	0,535	-1,929	3,695	0,003
Age at day of surgery	-0,331	0,503	-0,659	0,511	-1,327	0,665	0,004
Hemoglobin [mmol/l] prior to surgery	11,991	5,624	2,132	**0,035**	0,855	23,127	0,037
Lactate level [mmol/l] prior to surgery	-3,979	7,889	-0,504	0,615	-19,601	11,642	0,002
procalcotinin level [ng/ml] prior to surgery	1,146	1,532	0,748	0,456	-1,887	4,179	0,005
creatinine level [µmol/l] prior to surgery	-0,013	0,065	-0,198	0,843	-0,142	0,116	0,000
bilirubin level [µmol/l] prior to surgery	0,167	0,461	0,362	0,718	-0,746	1,079	0,001
WBC [Gpt/l] prior to surgery	-2,871	1,286	-2,233	0,027	-5,418	-0,325	0,041
platelet count [Gpt/l] prior to surgery	0,117	0,053	2,216	**0,029**	0,012	0,221	0,040
CRP level [mg/l] prior to surgery	-0,098	0,149	-0,660	0,511	-0,392	0,196	0,004
CRP level [mg/l] after surgery	0,221	0,198	1,118	0,266	-0,170	0,613	0,010
CRP level [mg/l] POD1	0,510	0,104	4,892	**0,000**	0,304	0,717	0,169

Dependent variable: **CRP POD3**

Parameter	Regressioncoefficient B	Standarderror	T	Sig.	Confidenceintervall		partial Eta-Quadrat
					lower limit	upper limit	
Constant Term	-15,956	33,713	-0,473	0,637	-82,860	50,947	0,002
ICS - yes	1,345	8,168	0,165	0,869	-14,863	17,554	0,000
male gender	-4,944	8,628	-0,573	0,568	-22,066	12,178	0,003
Reoperation - no	13,955	9,719	1,436	0,154	-5,332	33,243	0,021
Type of surgery - single AV sternotomy	11,582	12,858	0,901	0,370	-13,933	37,098	0,008
Type of surgery - single MV sternotomy	19,603	14,262	1,374	0,172	-8,700	47,906	0,019
Type of surgery - complex case	9,200	12,487	0,737	0,463	-15,579	33,979	0,006
Euroscore on admission	0,465	0,971	0,478	0,633	-1,463	2,392	0,002
Age at day of surgery	0,516	0,340	1,518	0,132	-0,158	1,190	0,023
Hemoglobin [mmol/l] prior to surgery	-0,269	3,989	-0,067	0,946	-8,184	7,646	0,000
Lactate level [mmol/l] prior to surgery	-13,616	5,759	-2,364	**0,020**	-25,044	-2,188	0,054
procalcotinin level [ng/ml] prior to surgery	-1,419	0,993	-1,429	0,156	-3,388	0,551	0,020
creatinine level [µmol/l] prior to surgery	0,041	0,042	0,961	0,339	-0,043	0,124	0,009
bilirubin level [µmol/l] prior to surgery	-0,156	0,305	-0,512	0,610	-0,763	0,450	0,003
WBC [Gpt/l] prior to surgery	-0,032	0,901	-0,036	0,972	-1,820	1,756	0,000
platelet count [Gpt/l] prior to surgery	-0,025	0,037	-0,677	0,500	-0,099	0,049	0,005
CRP level [mg/l] prior to surgery	0,238	0,110	2,156	**0,034**	0,019	0,457	0,045
CRP level [mg/l] after surgery	-0,213	0,146	-1,460	0,148	-0,502	0,077	0,021
CRP level [mg/l] POD1	-0,177	0,074	-2,390	**0,019**	-0,324	-0,030	0,055
CRP level [mg/l] POD2	0,935	0,061	15,427	**0,000**	0,815	1,055	0,708

Dependent variable: **WBC after surgery**

Parameter	Regressioncoefficient B	Standarderror	T	Sig.	Confidenceintervall		partial Eta-Quadrat
					lower limit	upper limit	
Constant Term	19,336	5,423	3,566	0,000	8,617	30,055	0,081
ICS - yes	2,962	1,300	2,277	**0,024**	0,391	5,532	0,035
male gender	0,226	1,430	0,158	0,874	-2,601	3,054	0,000
Reoperation - no	-0,466	1,638	-0,285	0,776	-3,703	2,770	0,001
Type of surgery - single AV sternotomy	-3,407	2,214	-1,539	0,126	-7,782	0,969	0,016
Type of surgery - single MV sternotomy	-4,196	2,457	-1,708	0,090	-9,052	0,660	0,020
Type of surgery - complex case	-2,705	2,142	-1,263	0,209	-6,940	1,529	0,011
Euroscore on admission	-0,284	0,160	-1,780	0,077	-0,600	0,031	0,022
Age at day of surgery	-0,003	0,054	-0,054	0,957	-0,111	0,105	0,000
Hemoglobin [mmol/l] prior to surgery	-1,048	0,620	-1,691	0,093	-2,273	0,177	0,019
Lactate level [mmol/l] prior to surgery	-1,470	0,584	-2,519	**0,013**	-2,624	-0,317	0,042
procalcotinin level [ng/ml] prior to surgery	0,116	0,150	0,775	0,439	-0,180	0,412	0,004
creatinine level [µmol/l] prior to surgery	0,001	0,007	0,082	0,935	-0,014	0,015	0,000
bilirubin level [µmol/l] prior to surgery	1,478E-05	0,050	0,000	1,000	-0,099	0,099	0,000
platelet count [Gpt/l] prior to surgery	0,006	0,006	1,072	0,286	-0,005	0,018	0,008
CRP level [mg/l] prior to surgery	-0,006	0,009	-0,692	0,490	-0,023	0,011	0,003
WBC [Gpt/l] prior to surgery	0,966	0,148	6,518	**0,000**	0,673	1,258	0,228

Dependent variable: **WBC POD1**

Parameter	Regressioncoefficient B	Standarderror	T	Sig.	[illegible] Confidenceintervall		partial Eta-Quadrat
					lower limit	upper limit	
Constant Term	1,007	4,407	0,229	0,820	-7,711	9,725	0,000
ICS - yes	1,429	1,054	1,356	0,178	-0,656	3,514	0,014
male gender	-0,488	1,129	-0,432	0,666	-2,721	1,745	0,001
Reoperation - no	1,064	1,266	0,840	0,402	-1,441	3,569	0,005
Type of surgery - single AV sternotomy	-2,406	1,706	-1,410	0,161	-5,780	0,969	0,015
Type of surgery - single MV sternotomy	-2,248	1,879	-1,197	0,234	-5,964	1,468	0,011
Type of surgery - complex case	-1,969	1,638	-1,202	0,231	-5,208	1,271	0,011
Euroscore on admission	0,045	0,127	0,352	0,726	-0,207	0,296	0,001
Age at day of surgery	0,031	0,042	0,740	0,461	-0,052	0,114	0,004
Hemoglobin [mmol/l] prior to surgery	0,035	0,497	0,070	0,944	-0,948	1,017	0,000
Lactate level [mmol/l] prior to surgery	-0,379	0,739	-0,512	0,609	-1,841	1,084	0,002
procalcotinin level [ng/ml] prior to surgery	-0,202	0,143	-1,411	0,161	-0,485	0,081	0,015
creatinine level [µmol/l] prior to surgery	0,008	0,006	1,408	0,162	-0,003	0,020	0,015
bilirubin level [µmol/l] prior to surgery	0,016	0,039	0,414	0,680	-0,061	0,093	0,001
platelet count [Gpt/l] prior to surgery	0,001	0,005	0,303	0,762	-0,008	0,011	0,001
CRP level [mg/l] prior to surgery	0,007	0,007	0,980	0,329	-0,007	0,020	0,007
WBC [Gpt/l] prior to surgery	0,384	0,131	2,940	**0,004**	0,126	0,643	0,061
WBC [Gpt/l] after surgery	0,384	0,064	5,952	**0,000**	0,256	0,511	0,212

Dependent variable: **WBC POD2**

Parameter	Regressioncoefficient B	Standarderror	T	Sig.	Confidenceintervall		partial Eta-Quadrat
					lower limit	upper limit	
Constant Term	-2,145	3,842	-0,558	0,578	-9,754	5,465	0,003
ICS - yes	0,227	0,896	0,253	0,801	-1,548	2,001	0,001
male gender	0,151	0,986	0,153	0,878	-1,802	2,105	0,000
Reoperation - no	-0,814	1,060	-0,768	0,444	-2,913	1,285	0,005
Type of surgery - single AV sternotomy	0,389	1,480	0,263	0,793	-2,542	3,320	0,001
Type of surgery - single MV sternotomy	0,723	1,687	0,428	0,669	-2,619	4,064	0,002
Type of surgery - complex case	0,485	1,434	0,338	0,736	-2,355	3,324	0,001
Euroscore on admission	0,062	0,109	0,567	0,572	-0,154	0,277	0,003
Age at day of surgery	0,038	0,038	1,003	0,318	-0,037	0,114	0,009
Hemoglobin [mmol/l] prior to surgery	0,499	0,439	1,137	0,258	-0,371	1,368	0,011
Lactate level [mmol/l] prior to surgery	0,250	0,607	0,412	0,681	-0,952	1,453	0,001
procalcotinin level [ng/ml] prior to surgery	0,151	0,117	1,291	0,199	-0,080	0,382	0,014
creatinine level [µmol/l] prior to surgery	-0,004	0,005	-0,817	0,415	-0,014	0,006	0,006
bilirubin level [µmol/l] prior to surgery	0,099	0,035	2,808	**0,006**	0,029	0,168	0,064
platelet count [Gpt/l] prior to surgery	-0,001	0,004	-0,368	0,713	-0,009	0,006	0,001
CRP level [mg/l] prior to surgery	0,006	0,006	1,021	0,310	-0,006	0,018	0,009
WBC [Gpt/l] prior to surgery	0,043	0,114	0,379	0,705	-0,183	0,269	0,001
WBC [Gpt/l] after surgery	0,018	0,067	0,270	0,787	-0,115	0,151	0,001
WBC [Gpt/l] POD1	0,581	0,084	6,883	**0,000**	0,414	0,748	0,290

Dependent variable: **WBC POD3**

Parameter	Regressioncoefficient B	Standarderror	T	Sig.	[illegible] Confidenceintervall		partial Eta-Quadrat
					lower limit	upper limit	
Constant Term	-0,356	3,597	-0,099	0,921	-7,498	6,785	0,000
ICS - yes	1,411	0,842	1,677	0,097	-0,259	3,082	0,029
male gender	-1,849	0,930	-1,989	**0,050**	-3,694	-0,004	0,040
Reoperation - no	-1,687	1,004	-1,680	0,096	-3,679	0,306	0,029
Type of surgery - single AV sternotomy	-0,164	1,337	-0,122	0,903	-2,817	2,490	0,000
Type of surgery - single MV sternotomy	0,155	1,539	0,101	0,920	-2,900	3,210	0,000
Type of surgery - complex case	0,350	1,298	0,270	0,788	-2,227	2,927	0,001
Euroscore on admission	-0,065	0,101	-0,646	0,520	-0,267	0,136	0,004
Age at day of surgery	0,010	0,035	0,290	0,772	-0,059	0,079	0,001
Hemoglobin [mmol/l] prior to surgery	0,049	0,417	0,118	0,907	-0,779	0,878	0,000
Lactate level [mmol/l] prior to surgery	0,790	0,598	1,321	0,190	-0,397	1,977	0,018
procalcotinin level [ng/ml] prior to surgery	-0,134	0,103	-1,300	0,197	-0,338	0,071	0,017
creatinine level [µmol/l] prior to surgery	-0,001	0,004	-0,145	0,885	-0,009	0,008	0,000
bilirubin level [µmol/l] prior to surgery	0,006	0,032	0,180	0,858	-0,059	0,070	0,000
platelet count [Gpt/l] prior to surgery	0,001	0,004	0,331	0,742	-0,007	0,009	0,001
CRP level [mg/l] prior to surgery	0,010	0,005	1,800	0,075	-0,001	0,021	0,033
WBC [Gpt/l] prior to surgery	0,129	0,104	1,241	0,218	-0,077	0,335	0,016
WBC [Gpt/l] after surgery	-0,024	0,065	-0,372	0,711	-0,153	0,105	0,001
WBC [Gpt/l] POD1	0,225	0,094	2,395	**0,019**	0,038	0,411	0,057
WBC [Gpt/l] POD2	0,683	0,083	8,269	**0,000**	0,519	0,848	0,419

Dependent variable: **VIS intraoperative**

Parameter	Regressioncoefficient B	Standarderror	T	Sig.	Confidenceintervall		partial Eta-Quadrat
					lower limit	upper limit	
Constant Term	-28,359	65,103	-0,436	0,664	-157,048	100,330	0,001
ICS - yes	25,707	13,893	1,850	0,066	-1,756	53,170	0,023
male gender	-18,630	15,028	-1,240	0,217	-48,336	11,075	0,011
Reoperation - no	-6,719	17,910	-0,375	0,708	-42,121	28,683	0,001
Type of surgery - single AV sternotomy	33,718	27,858	1,210	0,228	-21,349	88,784	0,010
Type of surgery - single MV sternotomy	10,147	29,150	0,348	0,728	-47,473	67,767	0,001
Type of surgery - complex case	15,255	27,529	0,554	0,580	-39,161	69,670	0,002
BMI [kg/m^2]	-0,949	1,342	-0,707	0,481	-3,602	1,704	0,003
Euroscore on admission	-2,493	1,618	-1,541	0,126	-5,691	0,705	0,016
CPB duration [minutes]	0,380	0,216	1,754	0,082	-0,048	0,807	0,021
CX duration [minutes]	-0,179	0,290	-0,617	0,538	-0,753	0,395	0,003
CPB reperfusion time [minutes]	0,289	0,403	0,717	0,475	-0,507	1,085	0,004
Age at day of surgery	-0,181	0,580	-0,312	0,755	-1,329	0,966	0,001
Lactate level [mmol/l] prior to surgery	19,725	5,776	3,415	**0,001**	8,307	31,143	0,075
procalcotinin level [ng/ml] prior to surgery	-0,617	0,884	-0,699	0,486	-2,365	1,130	0,003
creatinine level [µmol/l] prior to surgery	0,295	0,076	3,889	**0,000**	0,145	0,445	0,096
bilirubin level [µmol/l] prior to surgery	-0,654	0,529	-1,235	0,219	-1,700	0,393	0,011
CRP level [mg/l] prior to surgery	0,258	0,090	2,876	**0,005**	0,081	0,436	0,055
WBC [Gpt/l] prior to surgery	0,495	1,551	0,319	0,750	-2,570	3,560	0,001
Hemoglobin [mmol/l] prior to surgery	-16,305	27,536	-0,592	0,555	-70,736	38,126	0,002
hematocrit [%] prior to surgery	306,157	586,699	0,522	0,603	-853,566	1465,880	0,002
platelet count [Gpt/l] prior to surgery	0,076	0,061	1,250	0,213	-0,044	0,195	0,011

Dependent variable: **VIS after surgery**

Parameter	Regressioncoefficient B	Standarderror	T	Sig.	% Confidenceintervall		partial Eta-Quadrat
					lower limit	upper limit	
Constant Term	10,310	20,886	0,494	0,622	-30,977	51,598	0,002
ICS - yes	6,529	4,507	1,449	0,150	-2,381	15,439	0,015
male gender	-1,393	4,844	-0,288	0,774	-10,969	8,182	0,001
Reoperation - no	5,220	5,745	0,909	0,365	-6,136	16,576	0,006
Type of surgery - single AV sternotomy	2,422	8,977	0,270	0,788	-15,324	20,167	0,001
Type of surgery - single MV sternotomy	1,291	9,349	0,138	0,890	-17,191	19,773	0,000
Type of surgery - complex case	-1,247	8,835	-0,141	0,888	-18,713	16,218	0,000
BMI [kg/m^2]	-0,426	0,431	-0,989	0,324	-1,279	0,426	0,007
Euroscore on admission	0,340	0,523	0,651	0,516	-0,693	1,374	0,003
CPB duration [minutes]	-0,002	0,070	-0,024	0,981	-0,140	0,137	0,000
CX duration [minutes]	0,001	0,093	0,006	0,995	-0,184	0,185	0,000
CPB reperfusion time [minutes]	0,036	0,129	0,277	0,782	-0,220	0,292	0,001
Age at day of surgery	0,065	0,186	0,349	0,728	-0,303	0,433	0,001
Lactate level [mmol/l] prior to surgery	-1,946	1,926	-1,011	0,314	-5,753	1,861	0,007
procalcotinin level [ng/ml] prior to surgery	-0,500	0,284	-1,762	0,080	-1,061	0,061	0,021
creatinine level [µmol/l] prior to surgery	0,032	0,026	1,261	0,209	-0,018	0,083	0,011
bilirubin level [µmol/l] prior to surgery	0,317	0,171	1,858	0,065	-0,020	0,654	0,024
CRP level [mg/l] prior to surgery	-0,007	0,030	-0,248	0,804	-0,066	0,051	0,000
WBC [Gpt/l] prior to surgery	0,457	0,497	0,919	0,360	-0,526	1,440	0,006
Hemoglobin [mmol/l] prior to surgery	-5,208	8,839	-0,589	0,557	-22,681	12,265	0,002
hematocrit [%] prior to surgery	105,152	188,274	0,559	0,577	-267,031	477,334	0,002
platelet count [Gpt/l] prior to surgery	-0,033	0,020	-1,696	0,092	-0,072	0,005	0,020
VIS intraoperative	0,034	0,027	1,280	0,203	-0,019	0,087	0,011

Dependent variable: **VIS POD1**

Parameter	Regressioncoefficient B	Standarderror	T	Sig.	Confidenceintervall		partial Eta-Quadrat
					lower limit	upper limit	
Constant Term	0,000	6,134E-16	0,000	1,000	-1,213E-15	1,213E-15	0,000
ICS - yes	0,000	1,332E-16	0,000	1,000	-2,634E-16	2,634E-16	0,000
male gender	0,000	1,422E-16	0,000	1,000	-2,811E-16	2,811E-16	0,000
Reoperation - no	-8,882E-16	1,690E-16	-5,254	**0,000**	-1,222E-15	-5,540E-16	0,164
Type of surgery - single AV sternotomy	0,000	2,635E-16	0,000	1,000	-5,209E-16	5,209E-16	0,000
Type of surgery - single MV sternotomy	0,000	2,743E-16	0,000	1,000	-5,424E-16	5,424E-16	0,000
Type of surgery - complex case	0,000	2,593E-16	0,000	1,000	-5,125E-16	5,125E-16	0,000
BMI [kg/m^2]	0,000	1,269E-17	0,000	1,000	-2,509E-17	2,509E-17	0,000
Euroscore on admission	-5,551E-17	1,537E-17	-3,612	**0,000**	-8,589E-17	-2,513E-17	0,085
CPB duration [minutes]	2,168E-19	2,057E-18	0,105	0,916	-3,850E-18	4,284E-18	0,000
CX duration [minutes]	0,000	2,736E-18	0,000	1,000	-5,408E-18	5,408E-18	0,000
CPB reperfusion time [minutes]	0,000	3,797E-18	0,000	1,000	-7,507E-18	7,507E-18	0,000
Age at day of surgery	0,000	5,464E-18	0,000	1,000	-1,080E-17	1,080E-17	0,000
Lactate level [mmol/l] prior to surgery	0,000	5,671E-17	0,000	1,000	-1,121E-16	1,121E-16	0,000
procalcotinin level [ng/ml] prior to surgery	0,000	8,420E-18	0,000	1,000	-1,665E-17	1,665E-17	0,000
creatinine level [µmol/l] prior to surgery	0,000	7,547E-19	0,000	1,000	-1,492E-18	1,492E-18	0,000
bilirubin level [µmol/l] prior to surgery	0,000	5,067E-18	0,000	1,000	-1,002E-17	1,002E-17	0,000
CRP level [mg/l] prior to surgery	0,000	8,693E-19	0,000	1,000	-1,719E-18	1,719E-18	0,000
WBC [Gpt/l] prior to surgery	0,000	1,464E-17	0,000	1,000	-2,893E-17	2,893E-17	0,000
Hemoglobin [mmol/l] prior to surgery	0,000	2,597E-16	0,000	1,000	-5,134E-16	5,134E-16	0,000
hematocrit [%] prior to surgery	0,000	5,530E-15	0,000	1,000	-1,093E-14	1,093E-14	0,000
platelet count [Gpt/l] prior to surgery	0,000	5,784E-19	0,000	1,000	-1,143E-18	1,143E-18	0,000
VIS intraoperative	0,000	7,912E-19	0,000	1,000	-1,564E-18	1,564E-18	0,000
VIS after surgery	1,000	2,462E-18	###########	0,000	1,000	1,000	1,000

Dependent variable: **VIS POD2**

Parameter	Regressioncoefficient B	Standarderror	T	Sig.	% Confidenceintervall		partial Eta-Quadrat
					lower limit	upper limit	
Constant Term	-5,814	12,512	-0,465	0,643	-30,549	18,922	0,002
ICS - yes	-1,805	2,718	-0,664	0,508	-7,177	3,568	0,003
male gender	1,762	2,900	0,607	0,545	-3,972	7,495	0,003
Reoperation - no	1,239	3,448	0,359	0,720	-5,579	8,056	0,001
Type of surgery - single AV sternotomy	2,971	5,374	0,553	0,581	-7,654	13,596	0,002
Type of surgery - single MV sternotomy	2,454	5,596	0,438	0,662	-8,610	13,518	0,001
Type of surgery - complex case	4,821	5,289	0,912	0,364	-5,635	15,276	0,006
BMI [kg/m^2]	-0,162	0,259	-0,624	0,534	-0,673	0,350	0,003
Euroscore on admission	-0,192	0,313	-0,613	0,541	-0,812	0,428	0,003
CPB duration [minutes]	0,046	0,042	1,106	0,271	-0,037	0,129	0,009
CX duration [minutes]	-0,073	0,056	-1,312	0,192	-0,184	0,037	0,012
CPB reperfusion time [minutes]	-0,006	0,077	-0,079	0,937	-0,159	0,147	0,000
Age at day of surgery	0,007	0,111	0,060	0,952	-0,214	0,227	0,000
Lactate level [mmol/l] prior to surgery	-0,855	1,157	-0,739	0,461	-3,142	1,432	0,004
procalcotinin level [ng/ml] prior to surgery	0,013	0,172	0,074	0,941	-0,327	0,352	0,000
creatinine level [µmol/l] prior to surgery	0,012	0,015	0,781	0,436	-0,018	0,042	0,004
bilirubin level [µmol/l] prior to surgery	-0,127	0,103	-1,228	0,221	-0,331	0,077	0,011
CRP level [mg/l] prior to surgery	0,011	0,018	0,634	0,527	-0,024	0,046	0,003
WBC [Gpt/l] prior to surgery	0,013	0,299	0,043	0,966	-0,577	0,603	0,000
Hemoglobin [mmol/l] prior to surgery	-1,372	5,297	-0,259	0,796	-11,844	9,100	0,000
hematocrit [%] prior to surgery	51,906	112,816	0,460	0,646	-171,123	274,935	0,001
platelet count [Gpt/l] prior to surgery	-0,007	0,012	-0,568	0,571	-0,030	0,017	0,002
VIS intraoperative	0,010	0,016	0,634	0,527	-0,022	0,042	0,003
VIS after surgery	0,713	0,050	14,187	**0,000**	0,613	0,812	0,588

Dependent variable: **VIS POD3**

Parameter	Regressioncoefficient B	Standarderror	T	Sig.	Confidenceintervall		partial Eta-Quadrat
					lower limit	upper limit	
Constant Term	-4,840	11,748	-0,412	0,681	-28,066	18,387	0,001
ICS - yes	0,250	2,554	0,098	0,922	-4,799	5,299	0,000
male gender	-2,570	2,725	-0,943	0,347	-7,956	2,817	0,006
Reoperation - no	5,385	3,237	1,664	0,098	-1,014	11,785	0,019
Type of surgery - single AV sternotomy	3,595	5,048	0,712	0,478	-6,385	13,575	0,004
Type of surgery - single MV sternotomy	4,257	5,254	0,810	0,419	-6,131	14,645	0,005
Type of surgery - complex case	3,798	4,977	0,763	0,447	-6,041	13,637	0,004
BMI [kg/m^2]	-0,434	0,243	-1,783	0,077	-0,915	0,047	0,022
Euroscore on admission	0,079	0,294	0,269	0,788	-0,503	0,661	0,001
CPB duration [minutes]	0,047	0,040	1,183	0,239	-0,031	0,125	0,010
CX duration [minutes]	-0,025	0,053	-0,471	0,638	-0,129	0,079	0,002
CPB reperfusion time [minutes]	-0,080	0,073	-1,102	0,272	-0,224	0,064	0,009
Age at day of surgery	0,002	0,105	0,021	0,983	-0,205	0,209	0,000
Lactate level [mmol/l] prior to surgery	0,082	1,088	0,076	0,940	-2,068	2,232	0,000
procalcotinin level [ng/ml] prior to surgery	-0,071	0,161	-0,440	0,661	-0,390	0,248	0,001
creatinine level [µmol/l] prior to surgery	0,033	0,014	2,279	**0,024**	0,004	0,062	0,036
bilirubin level [µmol/l] prior to surgery	-0,006	0,097	-0,063	0,950	-0,199	0,187	0,000
CRP level [mg/l] prior to surgery	0,014	0,017	0,865	0,388	-0,019	0,047	0,005
WBC [Gpt/l] prior to surgery	-0,046	0,280	-0,164	0,870	-0,600	0,508	0,000
Hemoglobin [mmol/l] prior to surgery	0,861	4,971	0,173	0,863	-8,967	10,689	0,000
hematocrit [%] prior to surgery	7,535	105,926	0,071	0,943	-201,887	216,958	0,000
platelet count [Gpt/l] prior to surgery	-0,008	0,011	-0,690	0,491	-0,030	0,014	0,003
VIS intraoperative	-0,005	0,015	-0,351	0,726	-0,035	0,025	0,001
VIS after surgery	-0,191	0,073	-2,597	**0,010**	-0,336	-0,046	0,046
VIS POD2	0,948	0,079	11,997	**0,000**	0,792	1,104	0,507

Dependent variable: **Lactate after surgery**

Parameter	Regressioncoefficient B	Standarderror	T	Sig.	% Confidenceintervall		partial Eta-Quadrat
					lower limit	upper limit	
Constant Term	-2,016	2,395	-0,842	0,401	-6,750	2,718	0,005
ICS - yes	0,888	0,507	1,752	0,082	-0,114	1,890	0,021
male gender	0,744	0,553	1,344	0,181	-0,350	1,838	0,013
Reoperation - no	0,713	0,647	1,101	0,273	-0,567	1,992	0,009
Type of surgery - single AV sternotomy	-1,532	1,012	-1,514	0,132	-3,533	0,469	0,016
Type of surgery - single MV sternotomy	-0,847	1,052	-0,805	0,422	-2,927	1,234	0,005
Type of surgery - complex case	-1,749	0,995	-1,757	0,081	-3,717	0,219	0,022
BMI [kg/m^2]	0,091	0,049	1,857	0,065	-0,006	0,189	0,024
Euroscore on admission	0,036	0,060	0,605	0,546	-0,082	0,154	0,003
CPB duration [minutes]	0,009	0,008	1,155	0,250	-0,006	0,025	0,009
CX duration [minutes]	0,013	0,010	1,205	0,230	-0,008	0,033	0,010
CPB reperfusion time [minutes]	0,017	0,015	1,139	0,256	-0,012	0,046	0,009
Age at day of surgery	0,006	0,021	0,269	0,789	-0,036	0,047	0,001
Lactate level [mmol/l] prior to surgery	0,276	0,221	1,250	0,213	-0,160	0,712	0,011
procalcotinin level [ng/ml] prior to surgery	-0,094	0,056	-1,673	0,096	-0,205	0,017	0,020
creatinine level [µmol/l] prior to surgery	0,003	0,003	0,989	0,324	-0,003	0,009	0,007
bilirubin level [µmol/l] prior to surgery	-0,009	0,019	-0,443	0,658	-0,047	0,030	0,001
CRP level [mg/l] prior to surgery	0,002	0,003	0,612	0,542	-0,005	0,009	0,003
WBC [Gpt/l] prior to surgery	0,040	0,056	0,718	0,474	-0,071	0,151	0,004
Hemoglobin [mmol/l] prior to surgery	-1,075	0,995	-1,080	0,282	-3,042	0,892	0,008
hematocrit [%] prior to surgery	17,413	21,186	0,822	0,413	-24,472	59,298	0,005
platelet count [Gpt/l] prior to surgery	-0,004	0,002	-1,863	0,064	-0,009	0,000	0,024
VIS intraoperative	0,016	0,004	4,129	**0,000**	0,008	0,023	0,109

Dependent variable: **Lactate POD1**

Parameter	Regressioncoefficient B	Standarderror	T	Sig.	Confidenceintervall		partial Eta-Quadrat
					lower limit	upper limit	
Constant Term	-3,927	3,876	-1,013	0,313	-11,597	3,743	0,008
ICS - yes	-0,860	0,867	-0,992	0,323	-2,575	0,855	0,008
male gender	0,823	0,874	0,942	0,348	-0,906	2,551	0,007
Reoperation - no	-0,751	1,015	-0,740	0,461	-2,759	1,257	0,004
Type of surgery - single AV sternotomy	0,147	1,636	0,090	0,929	-3,091	3,384	0,000
Type of surgery - single MV sternotomy	0,959	1,674	0,573	0,568	-2,354	4,272	0,003
Type of surgery - complex case	0,854	1,610	0,530	0,597	-2,333	4,040	0,002
BMI [kg/m^2]	-0,028	0,079	-0,349	0,728	-0,184	0,129	0,001
Euroscore on admission	-0,122	0,095	-1,275	0,205	-0,310	0,067	0,013
CPB duration [minutes]	0,001	0,013	0,058	0,954	-0,024	0,026	0,000
CX duration [minutes]	0,003	0,017	0,188	0,851	-0,030	0,036	0,000
CPB reperfusion time [minutes]	-0,033	0,024	-1,352	0,179	-0,082	0,015	0,014
Age at day of surgery	0,103	0,033	3,121	**0,002**	0,038	0,168	0,071
Lactate level [mmol/l] prior to surgery	-0,090	0,357	-0,254	0,800	-0,796	0,615	0,001
procalcotinin level [ng/ml] prior to surgery	-0,041	0,088	-0,467	0,641	-0,214	0,133	0,002
creatinine level [µmol/l] prior to surgery	-0,004	0,005	-0,795	0,428	-0,013	0,006	0,005
bilirubin level [µmol/l] prior to surgery	-0,033	0,030	-1,098	0,274	-0,094	0,027	0,009
CRP level [mg/l] prior to surgery	0,001	0,006	0,232	0,817	-0,010	0,012	0,000
WBC [Gpt/l] prior to surgery	0,068	0,089	0,772	0,442	-0,107	0,244	0,005
Hemoglobin [mmol/l] prior to surgery	2,588	1,578	1,640	0,103	-0,535	5,712	0,021
hematocrit [%] prior to surgery	-60,556	33,784	-1,792	0,075	-127,404	6,292	0,024
platelet count [Gpt/l] prior to surgery	0,004	0,004	1,217	0,226	-0,003	0,012	0,011
VIS intraoperative	-0,012	0,008	-1,472	0,143	-0,028	0,004	0,017
Lactate level [mmol/l] after surgery	1,453	0,189	7,682	**0,000**	1,079	1,827	0,316
VIS after surgery	0,087	0,017	5,135	**0,000**	0,054	0,121	0,171

Dependent variable: **Lactate POD2**

Parameter	Regressioncoefficient B	Standarderror	T	Sig.	Confidenceintervall		partial Eta-Quadrat
					lower limit	upper limit	
Constant Term	0,739	2,094	0,353	0,725	-3,410	4,888	0,001
ICS - yes	-0,062	0,485	-0,128	0,898	-1,024	0,899	0,000
male gender	-0,228	0,475	-0,479	0,633	-1,170	0,715	0,002
Reoperation - no	-0,412	0,548	-0,753	0,453	-1,498	0,673	0,005
Type of surgery - single AV sternotomy	-1,426	0,927	-1,538	0,127	-3,263	0,411	0,021
Type of surgery - single MV sternotomy	-1,739	0,968	-1,797	0,075	-3,656	0,179	0,028
Type of surgery - complex case	-1,686	0,920	-1,832	0,070	-3,510	0,138	0,029
BMI [kg/m^2]	0,040	0,045	0,885	0,378	-0,049	0,128	0,007
Euroscore on admission	0,017	0,053	0,318	0,751	-0,088	0,121	0,001
CPB duration [minutes]	-0,012	0,007	-1,758	0,082	-0,027	0,002	0,027
CX duration [minutes]	0,026	0,009	2,888	**0,005**	0,008	0,044	0,069
CPB reperfusion time [minutes]	0,006	0,013	0,450	0,653	-0,020	0,032	0,002
Age at day of surgery	0,003	0,020	0,150	0,881	-0,037	0,043	0,000
Lactate level [mmol/l] prior to surgery	-0,065	0,186	-0,346	0,730	-0,434	0,305	0,001
procalcotinin level [ng/ml] prior to surgery	0,002	0,046	0,054	0,957	-0,088	0,093	0,000
creatinine level [µmol/l] prior to surgery	-0,005	0,002	-1,889	0,062	-0,009	0,000	0,031
bilirubin level [µmol/l] prior to surgery	0,035	0,018	1,970	**0,051**	0,000	0,071	0,034
CRP level [mg/l] prior to surgery	0,001	0,003	0,343	0,733	-0,005	0,007	0,001
WBC [Gpt/l] prior to surgery	-0,013	0,049	-0,276	0,783	-0,110	0,083	0,001
Hemoglobin [mmol/l] prior to surgery	-0,457	0,874	-0,523	0,602	-2,189	1,275	0,002
hematocrit [%] prior to surgery	8,165	18,449	0,443	0,659	-28,390	44,719	0,002
platelet count [Gpt/l] prior to surgery	-0,001	0,002	-0,735	0,464	-0,005	0,002	0,005
VIS intraoperative	-0,006	0,005	-1,319	0,190	-0,015	0,003	0,015
Lactate level [mmol/l] after surgery	0,257	0,151	1,706	0,091	-0,041	0,556	0,025
VIS after surgery	0,023	0,012	1,941	**0,055**	0,000	0,046	0,033
Lactate level [mmol/l] POD1 surgery	0,386	0,082	4,689	**0,000**	0,223	0,549	0,164

Dependent variable: **Lactate POD3**

Parameter	Regressioncoefficient B	Standarderror	T	Sig.	Confidenceintervall		partial Eta-Quadrat
					lower limit	upper limit	
Constant Term	1,574	0,984	1,599	0,113	-0,381	3,530	0,027
ICS - yes	0,514	0,231	2,230	**0,028**	0,056	0,972	0,052
male gender	-0,576	0,217	-2,662	**0,009**	-1,007	-0,146	0,072
Reoperation - no	0,218	0,268	0,814	0,418	-0,314	0,749	0,007
Type of surgery - single AV sternotomy	0,580	0,411	1,412	0,161	-0,236	1,396	0,021
Type of surgery - single MV sternotomy	0,862	0,440	1,956	**0,053**	-0,013	1,737	0,040
Type of surgery - complex case	0,768	0,407	1,888	0,062	-0,040	1,576	0,038
BMI [kg/m^2]	-0,064	0,020	-3,139	**0,002**	-0,105	-0,024	0,098
Euroscore on admission	-0,012	0,025	-0,500	0,618	-0,061	0,036	0,003
CPB duration [minutes]	0,002	0,003	0,653	0,515	-0,004	0,009	0,005
CX duration [minutes]	-0,004	0,004	-0,961	0,339	-0,012	0,004	0,010
CPB reperfusion time [minutes]	-0,010	0,006	-1,614	0,110	-0,022	0,002	0,028
Age at day of surgery	0,015	0,009	1,558	0,123	-0,004	0,033	0,026
Lactate level [mmol/l] prior to surgery	0,119	0,082	1,458	0,148	-0,043	0,281	0,023
procalcotinin level [ng/ml] prior to surgery	-0,031	0,025	-1,225	0,224	-0,081	0,019	0,016
creatinine level [µmol/l] prior to surgery	0,003	0,001	2,633	**0,010**	0,001	0,005	0,071
bilirubin level [µmol/l] prior to surgery	-0,006	0,008	-0,679	0,499	-0,022	0,011	0,005
CRP level [mg/l] prior to surgery	-0,002	0,001	-1,209	0,230	-0,005	0,001	0,016
WBC [Gpt/l] prior to surgery	-0,010	0,023	-0,458	0,648	-0,056	0,035	0,002
Hemoglobin [mmol/l] prior to surgery	0,357	0,380	0,938	0,351	-0,399	1,113	0,010
hematocrit [%] prior to surgery	-7,715	8,142	-0,947	0,346	-23,889	8,459	0,010
platelet count [Gpt/l] prior to surgery	-0,001	0,001	-0,715	0,476	-0,003	0,001	0,006
VIS intraoperative	0,000	0,002	-0,048	0,962	-0,005	0,004	0,000
Lactate level [mmol/l] after surgery	-0,082	0,085	-0,965	0,337	-0,252	0,087	0,010
VIS after surgery	-0,002	0,006	-0,412	0,681	-0,014	0,009	0,002
Lactate level [mmol/l] POD1 surgery	0,029	0,044	0,657	0,513	-0,059	0,117	0,005
Lactate level [mmol/l] POD2 surgery	0,503	0,046	10,899	**0,000**	0,411	0,594	0,566

Dependent variable: **SOFA after surgery**

Parameter	Regressioncoefficient B	Standarderror	T	Sig.	Confidenceintervall		partial Eta-Quadrat
					lower limit	upper limit	
Constant Term	4,650	1,406	3,308	0,001	1,869	7,430	0,076
ICS - yes	-0,065	0,279	-0,234	0,816	-0,618	0,487	0,000
male gender	-0,090	0,306	-0,295	0,768	-0,696	0,515	0,001
Reoperation - no	-0,343	0,357	-0,961	0,338	-1,048	0,363	0,007
Type of surgery - single AV sternotomy	-0,246	0,574	-0,429	0,668	-1,381	0,889	0,001
Type of surgery - single MV sternotomy	0,270	0,599	0,451	0,653	-0,915	1,455	0,002
Type of surgery - complex case	0,319	0,561	0,569	0,571	-0,791	1,429	0,002
BMI [kg/m^2]	-0,024	0,028	-0,856	0,394	-0,079	0,031	0,005
Euroscore on admission	-0,050	0,033	-1,502	0,136	-0,115	0,016	0,017
CPB duration [minutes]	0,000	0,005	-0,073	0,942	-0,009	0,009	0,000
CX duration [minutes]	0,003	0,006	0,546	0,586	-0,008	0,015	0,002
CPB reperfusion time [minutes]	0,001	0,008	0,135	0,893	-0,015	0,018	0,000
Age at day of surgery	0,021	0,011	1,857	0,066	-0,001	0,044	0,025
Lactate level [mmol/l] prior to surgery	-0,113	0,118	-0,962	0,338	-0,346	0,120	0,007
procalcotinin level [ng/ml] prior to surgery	-0,002	0,019	-0,105	0,916	-0,039	0,035	0,000
creatinine level [µmol/l] prior to surgery	0,004	0,002	2,525	**0,013**	0,001	0,008	0,046
bilirubin level [µmol/l] prior to surgery	0,032	0,011	2,958	**0,004**	0,011	0,053	0,062
CRP level [mg/l] prior to surgery	-0,001	0,002	-0,365	0,715	-0,004	0,003	0,001
WBC [Gpt/l] prior to surgery	0,083	0,031	2,623	**0,010**	0,020	0,145	0,049
Hemoglobin [mmol/l] prior to surgery	0,190	0,545	0,349	0,728	-0,888	1,268	0,001
hematocrit [%] prior to surgery	-6,666	11,596	-0,575	0,566	-29,603	16,270	0,002
platelet count [Gpt/l] prior to surgery	-0,006	0,001	-4,626	**0,000**	-0,008	-0,003	0,139
VIS intraoperative	0,003	0,002	1,582	0,116	-0,001	0,006	0,018
SOFA prior to surgery	0,157	0,080	1,960	**0,052**	-0,001	0,316	0,028
VIS after surgery	0,024	0,005	4,567	**0,000**	0,013	0,034	0,136

Dependent variable: **SOFA POD1**

Parameter	Regressioncoefficient B	Standarderror	T	Sig.	Confidenceintervall		partial Eta-Quadrat
					lower limit	upper limit	
Constant Term	-4,922	2,415	-2,038	0,044	-9,700	-0,144	0,032
ICS - yes	0,560	0,481	1,163	0,247	-0,393	1,512	0,011
male gender	-0,646	0,507	-1,273	0,205	-1,650	0,358	0,013
Reoperation - no	0,172	0,576	0,298	0,766	-0,969	1,312	0,001
Type of surgery - single AV sternotomy	-0,474	0,933	-0,508	0,613	-2,320	1,372	0,002
Type of surgery - single MV sternotomy	0,001	0,966	0,001	0,999	-1,911	1,913	0,000
Type of surgery - complex case	-0,538	0,904	-0,595	0,553	-2,327	1,252	0,003
BMI [kg/m^2]	0,035	0,045	0,769	0,443	-0,055	0,124	0,005
Euroscore on admission	0,027	0,055	0,488	0,626	-0,081	0,135	0,002
CPB duration [minutes]	-0,002	0,007	-0,228	0,820	-0,017	0,013	0,000
CX duration [minutes]	0,012	0,009	1,237	0,218	-0,007	0,030	0,012
CPB reperfusion time [minutes]	0,004	0,014	0,303	0,763	-0,024	0,033	0,001
Age at day of surgery	0,048	0,019	2,509	0,013	0,010	0,085	0,047
Lactate level [mmol/l] prior to surgery	-0,061	0,194	-0,314	0,754	-0,445	0,323	0,001
procalcotinin level [ng/ml] prior to surgery	0,014	0,032	0,443	0,658	-0,049	0,078	0,002
creatinine level [µmol/l] prior to surgery	-0,001	0,003	-0,263	0,793	-0,006	0,005	0,001
bilirubin level [µmol/l] prior to surgery	0,039	0,018	2,165	**0,032**	0,003	0,075	0,036
CRP level [mg/l] prior to surgery	0,000	0,003	0,050	0,960	-0,006	0,006	0,000
WBC [Gpt/l] prior to surgery	-0,006	0,053	-0,104	0,917	-0,111	0,100	0,000
Hemoglobin [mmol/l] prior to surgery	0,383	0,892	0,430	0,668	-1,381	2,147	0,001
hematocrit [%] prior to surgery	-6,018	18,931	-0,318	0,751	-43,480	31,444	0,001
platelet count [Gpt/l] prior to surgery	0,000	0,002	-0,202	0,840	-0,005	0,004	0,000
VIS intraoperative	0,002	0,003	0,659	0,511	-0,004	0,008	0,003
SOFA prior to surgery	-0,116	0,132	-0,878	0,382	-0,376	0,145	0,006
VIS after surgery	0,037	0,009	3,982	**0,000**	0,019	0,055	0,111
SOFA after surgery	0,844	0,152	5,567	**0,000**	0,544	1,143	0,196

Dependent variable: **SOFA POD2**

Parameter	Regressioncoefficient B	Standarderror	T	Sig.	Confidenceintervall		partial Eta-Quadrat
					lower limit	upper limit	
Constant Term	4,347	2,663	1,632	0,105	-0,928	9,622	0,023
ICS - yes	-0,100	0,564	-0,177	0,860	-1,216	1,017	0,000
male gender	-1,256	0,575	-2,187	0,031	-2,394	-0,118	0,040
Reoperation - no	-0,228	0,639	-0,356	0,723	-1,494	1,039	0,001
Type of surgery - single AV sternotomy	1,321	1,057	1,250	0,214	-0,772	3,413	0,013
Type of surgery - single MV sternotomy	0,530	1,086	0,488	0,626	-1,621	2,680	0,002
Type of surgery - complex case	1,760	1,020	1,726	0,087	-0,260	3,781	0,025
BMI [kg/m^2]	0,035	0,052	0,666	0,507	-0,069	0,138	0,004
Euroscore on admission	-0,004	0,063	-0,068	0,946	-0,129	0,120	0,000
CPB duration [minutes]	0,006	0,008	0,772	0,441	-0,010	0,022	0,005
CX duration [minutes]	-0,011	0,010	-1,042	0,300	-0,031	0,010	0,009
CPB reperfusion time [minutes]	0,002	0,016	0,142	0,887	-0,029	0,033	0,000
Age at day of surgery	-0,025	0,023	-1,086	0,280	-0,070	0,020	0,010
Lactate level [mmol/l] prior to surgery	0,119	0,211	0,563	0,574	-0,300	0,537	0,003
procalcotinin level [ng/ml] prior to surgery	0,013	0,035	0,370	0,712	-0,056	0,082	0,001
creatinine level [µmol/l] prior to surgery	0,000	0,003	0,040	0,968	-0,006	0,006	0,000
bilirubin level [µmol/l] prior to surgery	-0,014	0,020	-0,667	0,506	-0,054	0,027	0,004
CRP level [mg/l] prior to surgery	-0,002	0,003	-0,597	0,551	-0,009	0,005	0,003
WBC [Gpt/l] prior to surgery	0,078	0,060	1,300	0,196	-0,041	0,197	0,014
Hemoglobin [mmol/l] prior to surgery	-0,123	0,993	-0,124	0,902	-2,090	1,844	0,000
hematocrit [%] prior to surgery	0,627	20,653	0,030	0,976	-40,282	41,536	0,000
platelet count [Gpt/l] prior to surgery	-0,006	0,003	-2,441	**0,016**	-0,012	-0,001	0,049
VIS intraoperative	-0,004	0,003	-1,115	0,267	-0,010	0,003	0,011
SOFA prior to surgery	-0,166	0,148	-1,127	0,262	-0,459	0,126	0,011
VIS after surgery	0,011	0,017	0,638	0,525	-0,023	0,045	0,004
SOFA after surgery	-0,185	0,187	-0,990	0,324	-0,555	0,185	0,008
VIS POD1	0,701	0,099	7,083	**0,000**	0,505	0,897	0,304
SOFA POD1	0,021	0,016	1,249	0,214	-0,012	0,053	0,013

Dependent variable: **SOFA POD3**

Parameter	Regressioncoefficient B	Standarderror	T	Sig.	Confidenceintervall		partial Eta-Quadrat
					lower limit	upper limit	
Constant Term	-0,478	1,920	-0,249	0,804	-4,287	3,331	0,001
ICS - yes	-0,116	0,406	-0,286	0,775	-0,921	0,689	0,001
male gender	0,669	0,418	1,600	0,113	-0,160	1,499	0,024
Reoperation - no	0,524	0,468	1,119	0,266	-0,404	1,452	0,012
Type of surgery - single AV sternotomy	-0,171	0,751	-0,228	0,820	-1,661	1,318	0,001
Type of surgery - single MV sternotomy	0,248	0,769	0,322	0,748	-1,277	1,772	0,001
Type of surgery - complex case	-0,476	0,735	-0,648	0,519	-1,933	0,981	0,004
BMI [kg/m^2]	0,037	0,038	0,961	0,339	-0,039	0,113	0,009
Euroscore on admission	0,004	0,046	0,084	0,933	-0,088	0,096	0,000
CPB duration [minutes]	0,006	0,006	0,944	0,347	-0,007	0,019	0,009
CX duration [minutes]	-0,010	0,008	-1,247	0,215	-0,026	0,006	0,015
CPB reperfusion time [minutes]	0,005	0,012	0,414	0,680	-0,019	0,029	0,002
Age at day of surgery	-0,002	0,017	-0,145	0,885	-0,035	0,031	0,000
Lactate level [mmol/l] prior to surgery	0,008	0,150	0,052	0,958	-0,290	0,305	0,000
procalcotinin level [ng/ml] prior to surgery	-0,027	0,025	-1,067	0,288	-0,076	0,023	0,011
creatinine level [µmol/l] prior to surgery	0,000	0,002	-0,108	0,914	-0,005	0,004	0,000
bilirubin level [µmol/l] prior to surgery	0,008	0,017	0,475	0,636	-0,025	0,041	0,002
CRP level [mg/l] prior to surgery	0,002	0,003	0,880	0,381	-0,003	0,007	0,008
WBC [Gpt/l] prior to surgery	-0,009	0,043	-0,207	0,837	-0,095	0,077	0,000
Hemoglobin [mmol/l] prior to surgery	0,212	0,715	0,296	0,768	-1,207	1,630	0,001
hematocrit [%] prior to surgery	-5,935	15,053	-0,394	0,694	-35,792	23,923	0,002
platelet count [Gpt/l] prior to surgery	0,000	0,002	-0,255	0,799	-0,004	0,003	0,001
VIS intraoperative	0,001	0,002	0,222	0,825	-0,004	0,005	0,000
SOFA prior to surgery	-0,030	0,111	-0,272	0,786	-0,250	0,189	0,001
VIS after surgery	0,012	0,013	0,931	0,354	-0,014	0,039	0,008
SOFA after surgery	-0,025	0,136	-0,186	0,853	-0,295	0,245	0,000
SOFA POD1	0,003	0,090	0,028	0,978	-0,176	0,181	0,000
VIS POD2	-0,058	0,018	-3,291	**0,001**	-0,093	-0,023	0,096
SOFA POD2	0,756	0,078	9,707	**0,000**	0,601	0,910	0,480
VIS POD3	0,089	0,012	7,294	**0,000**	0,065	0,113	0,343
